# Our Delay

**Published:** 1834

**Authors:** 


### II.—ORIGINAL.

### The Western Journal.

*E Sylvas Nuncius.*

CINCINNATI, JANUARY, 1835.

### I. OUR DELAY.

The delay of a month in the publication of this number, requires an explanation if not an apology. Let us to it then. Well, in two of our preceding numbers, we announced that on the 5th of January, 1835, our brethren of Ohio, were to hold, in the Capitol, a great Convention, and, very naturally concluding, that it would do and say many good things, we felt anxious to give utterance to them, through our quarterly, at the earliest convenient period, and so we kept open our editorial department, that we might publish an abstract of the proceedings. An abstract was all that we at first intended; but as the Convention exerted itself on but few subjects, which are not of general interest, we have thought it would, on the whole, be more acceptable to our readers, to give the journal entire, which we do, accordingly, in the next article. It is printed from proof sheets of the pamphlet about to be issued by Drs. Parsons, Rives and Reed, the publishing committee of the Convention, who have obligingly favoured us with the means of making this early report of its proceedings.

